# Sexting Behaviors Before and During COVID-19 in Italian and Colombian Young Adults

**DOI:** 10.1007/s13178-023-00798-z

**Published:** 2023-03-06

**Authors:** Mara Morelli, Maryluz Gomez Plata, Stefano Isolani, Maryoris Elena Zapata Zabala, Kattia Paola Cabas Hoyos, Liliana Maria Uribe Tirado, Marcela Sucel Ruiz Gracia, Carmelina Paba Barbosa, Jessica Pistella, Antonio Zuffianò, Maria Gerbino, Fiorenzo Laghi, Concetta Pastorelli, Roberto Baiocco

**Affiliations:** 1 Department of Dynamic and Clinical Psychology, and Health Studies, Sapienza University of Rome, Rome, Italy; 2 Universidad del Magdalena, Santa Marta, Colombia; 3 Department of Developmental and Social Psychology, Faculty of Medicine and Psychology, Sapienza University of Rome, Rome, Italy; 4 Universidad de San Buenaventura, Medellín, Colombia; 5 Department of Psychology, Sapienza University of Rome, Rome, Italy

**Keywords:** Sexting, Online sexual activities, Pandemic-related stress, COVID-19 pandemic, Cross-countries study, Sexual orientation

## Abstract

**Introduction:**

Recent research highlight increasing at-risk online sexual activities and behaviors during the COVID-19 pandemic among young adults. Specifically, sexting refers to exchanging sexually suggestive messages, photos, and videos through technological devices, and it can be placed on a continuum from safer to riskier behavior. This study aims to improve our knowledge about sexting behaviors in Italian and Colombian young adults before and during the COVID-19 pandemic.

**Methods:**

A survey online was filled in by all recruited participants through a snowball sampling procedure (from December 2019 to June 2021) both in Italy and Colombia, resulting in a total of 2931 participants (2051 from Italy and 880 from Colombia) aged 18 to 35 years old (*M*_age_ = 23.85; *SD*_age_ = 3.63; 67.6% girls).

**Results:**

Italian youth were more engaged in risky sexting than Colombian participants, while Colombians indicated being more engaged in non-consensual sexting than Italians. Sexual minority people reported more sharing their own sexts, higher levels of sexting under pressure, and 3.2 times more risky sexting than exclusively heterosexual counterparts. During the pandemic period, participants sent their own sexts 1.5 times more and were less involved in non-consensual sexting than in the pre-pandemic era.

**Conclusions:**

The present research could help understand better the cultural dynamics underlying the differences in sexting behaviors, suggesting the relevance of investigating how sexting behaviors and online at-risk activities have changed since the pandemic started.

**Policy Implications:**

Results provide implications for educational and prevention programs to improve young people's awareness of sexting behaviors.

## Introduction

The spread of technologies and Internet availability has increased during the last twenty years, modifying our way of communicating with others, including sexual communication. Although researchers disagree with its definition (e.g., text-based vs. image-based), with the term *sexting* (crasis of the word *sex* and *texting*), we refer to the exchange of sexually suggestive messages, photos, and videos (i.e., sexts) through technological devices (Chalfen, 2009). The literature on sexting seems to be divided between considering sexting as a new normal sexual behavior or deviant behavior (Döring, 2014).

Recent meta-analyses discussed various forms of sexting (Mori et al., 2019, 2020) that can be placed on a continuum from safer to riskier behavior. At the safer point of this continuum, we can find *experimental sexting:* a consensual exchange of sexts that can help people address their developmental tasks and needs related to the exploration and construction of their sexuality and identity (Bianchi et al., 2019; Kosenko et al., 2017; Morelli et al., 2021a). This concept was first introduced by Wolak and colleagues (2012), who considered sexting a new sexual normative behavior, especially during adolescence and young adulthood, developmental phases in which individuals explore their sexuality and the adequacy of their body image (Bianchi et al., 2021b; Morelli et al., 2017b).

Experimental sexing has been associated with passion, intimacy, and fun (Drouin & Tobin, 2014; Van Ouytsel et al., 2019) and with higher communication in sexual minorities (Chong et al., 2015). Moreover, several studies have highlighted its role in increasing self-esteem and reinforcing one’s body image (Bianchi et al., 2017). Experimental sexting can also be used for sexual purposes (Bianchi et al., 2021b), such as flirting (Albury & Crawford, 2012), maintaining a relationship (Van Ouytsel et al., 2017), or initiating sexual activities (Temple, 2015). It is commonly considered a way to increase passion and intimacy (Parker et al., 2013), especially in long-distance relationships (Walker et al., 2013).

On the other hand, sexting may have a negative side (Van Ouytsel et al., 2020) and can be used for different purposes, such as earning money (Morelli et al., 2017b) or responding to pressure from others (Currin & Hubach, 2019). For instance, in a recent study (Bianchi et al., 2021a, b, c), sexting for secondary aims, such as obtaining favors or money, and sexting for harmful intentions, was found to be a risk factor for dating violence perpetration and victimization.

Indeed, on the negative side of the sexting behavior continuum, there is *aggravated sexting* in which there are harmful intentions underlying sexting. Specifically, aggravated sexting covers non-consensual sexting, that is forwarding someone’s sexts without their permission (i.e., a dimension more related to perpetration of violence; Morelli et al., 2016a) and forced sexting, which is the coercion of sexting under threats or pressure of partners or friends (i.e., a dimension more related to victimization; Drouin & Tobin, 2014). Aggravated sexting can have several negative consequences, such as bullying, cyberbullying, and dating violence (Bianchi et al., 2021c; Gámez-Guadix et al., 2019; Gassó et al., 2019, 2021; Morelli et al., 2017b).

One last kind of sexting can be considered *risky sexting*: this kind of sexting does not imply a coercive dynamic, which includes a perpetrator/victim role of the sexters, but the co-occurrence of risky behaviors and sexting, such as engaging in sexting behaviors under the influence of drugs, alcohol, or sharing sexts with strangers. There are inconsistent results in the literature regarding the relationship between sexting and risky behaviors (e.g., alcohol or substance use). Indeed, a recent meta-analysis showed inconsistent findings on the relationship between sexting and risky behaviors (Kosenko et al., 2017), but another recent systematic review suggests a high association between them (Mori et al., 2019). Some studies have found a relationship between sexting and substance use (Morelli et al., 2017a) and the use of several recreational drugs, such as alcohol, ecstasy, marijuana, and cocaine (Benotsch et al., 2013).

## Age, Gender, and Sexual Orientation Differences in Sexting

Literature shed light on age differences in sexting behaviors; in particular, young adults showed a higher prevalence than adolescents (Bianchi et al., 2019; Madigan et al., 2018). Indeed, it is during young adulthood that sexting behaviors reach their peak (Mori et al., 2020), probably because during this life stage, people are still in a phase of exploration of sexuality and romantic relationships (Morgan, 2013). Thus, young people can be more inclined to explore different sexual behaviors, such as sexting (Mori et al., 2020), with both committed and casual partners (Brodie et al., 2019). Indeed, the prevalence of sexts sent in a committed relationship seems to be about 60%, and about 44% with a casual partner (Drouin et al., 2017). In particular, young adults in a dating relationship report higher percentages of experimental and risky sexting than their pairs not involved in a relationship (Morelli et al., 2020). In addition, the spread of technology is relatively recent, and young adults, also referred to as digital natives, are those who make the most use of it, even for personal reasons (Vergés Bosch et al., 2021).

A recent meta-analysis (Mori et al., 2020) reported that among young adults, the prevalence of sending sexts ranged between 32 and 44.6%, and receiving sexts between 31.9 and 51.2%. Moreover, a recent cross-cultural study (Morelli et al., 2020) has shown that both experimental and risky sexting increase with age. Regarding gender differences, there are inconsistent results in the literature. Some studies found higher percentages of generic sexting behaviors in females (Van Ouytsel et al., 2017) and other in males (Hudson & Marshall, 2016), while the meta-analysis by Madigan and colleagues (2018) have found no gender differences in sexting behaviors.

Moreover, Morelli and colleagues (2020) found a higher percentage of aggravated and risky sexting in young adult males than in females. Finally, regarding sexual orientation differences, sexual minorities young adults seem to be more likely to engage in sexting (Bianchi et al., 2019; Morelli et al., 2020; Van Ouytsel et al., 2019), probably because the virtual environment makes them feel protected by prejudice and sexual stigma they are exposed to as theorized by Meyer (2003) in the Minority Stress Model (Chong et al., 2015).

## Sexting and COVID-19 Pandemic

COVID-19 pandemic has strongly influenced people’s life (Armour et al., 2021; Babore et al., 2021, 2021b; Morelli et al., 2021b; Pistella et al., 2022; Trumello et al., 2021) and sexual behaviors (Bianchi et al., 2021a; Döring, 2020; Eleuteri & Terzitta, 2021). Together and successively with the stay-at-home laws, public health institutions proclaimed the need to avoid physical and sexual contact, suggesting online sexual activities as a safer way to contrast COVID-19 contagion (e.g., International Society for the Study of Women’s Sexual Health, 2020). Therefore, virtual sexual behaviors such as sexting were considered the best option during and after lockdown (Bianchi et al., 2021a).

Moreover, the fear of contagions and home confinement have motivated people to engage in these behaviors in different ways. Firstly, couples not cohabiting were forced into a long-distance relationship (Wijayanti, 2021), and sexting could have been the best alternative to maintain their intimacy and sexual desire (Bianchi et al., 2021a). Secondly, for people who used to be involved in many casual sexual relationships that were strongly forbidden during the pandemic (Wignall et al., 2021), sexting and other virtual sexual activities might have been the easiest and safest way to satisfy their sexual desires.

Indeed, during the lockdown, the increase of percentages reported for online sexual activities was between 28 and 38% (Ballester-Arnal et al., 2020; Gabster et al., 2021) and was associated with a reduction in traditional casual sex (Gabster et al., 2021). During this period, 15% of young adults started to sext, reporting increased satisfaction with their sexual life (Lehmiller et al., 2020). Moreover, Nelson and colleagues (2020) found an increase in sexting during pandemics among sexual minorities. Lesbian, gay, and bisexual (LGB) young adults could have been more distressed during lockdown due to the confinement with their families, who could be unwelcoming to their sexual orientation (Woznicki et al., 2020). Thus, LGB young adults could have used online communication to stay in contact with friends, look for emotional support, and express their sexuality (Bianchi et al., 2021a; Eleuteri & Terzitta, 2021; Woznicki et al., 2020).

## Cross-cultural Differences in Sexting

Sexual behaviors are strongly influenced by society and culture (Worthen et al., 2017). Some sexual activities can be culturally encouraged or discouraged, controlling peoples’ expression of sexuality. For instance, more conservative politics are linked to less favorable attitudes regarding sexual activities (Worthen et al., 2017), like sexting. Moreover, the culture of societies with stricter gender roles has a broad impact on sexual expression (Eisenman & Dantzker, 2006), and the presence of hostile sexist attitudes can be a risk factor for the forwarding of sexts without the consent of the person portrayed (Morelli et al., 2017a).

Morelli and colleagues (2020), in a cross-cultural study on ten different countries (i.e., Belgium, China, Czech Republic, Ireland, Italy, Poland, Russia, Turkey, Uganda, and the USA), reported that between 14.3 and 54.4% of participants had shared their own sexts at least once, between 12.4 and 53.2% engaged in risky sexting at least once, between 9 and 21.2% were involved in non-consensual sexting, and between 5.5 and 35.9% were involved in sexting under pressure.

In a recent study conducted by EU Kids Online (Smahel et al., 2020), which involved adolescents from 19 European countries, results have shown different sexting prevalences across different cultures. For instance, Italy and Slovakia, culturally diverse countries, reported similar prevalences for both sending (2% and 3%, respectively) and receiving (8% and 9%, respectively) sexts. On the other hand, Italy and Spain, culturally similar countries, reported different prevalence, with 30% of Spanish (vs. 2% of Italian) participants reporting having sent a sext and 9% (vs. 8% of Italian) reporting having received a sext.

To our knowledge, only one recent study by Gil-Lario and colleagues (2020) investigated sexting behaviors in Colombian adolescents, analyzing the prevalence of sexting between Spain and Colombia: Specifically, 28% of Spanish and 49% of Colombians reported to have been involved at least once in sexting. Gil-Lario et al. (2020) suggested that this difference was due to the role of some cultural influences on sexual behaviors. Indeed, Colombian adolescents seem to report a higher number of sexual behaviors together with an earlier sexual debut, if compared to Spanish adolescents. Another recent study on Colombian Caribbean university students (Gonzalez et al., 2021) found that young adults decrease their engagement in sexting between 18 and 20 years old and increase it between 20 and 22. Moreover, young men were more likely to be involved in sexting than young women. Thus, more studies are needed to understand better the influence of culture on sexting.

## Present Study

Sexting is a widespread phenomenon thanks to the ever-increasing diffusion of Internet connections and technological devices that offers opportunities to explore one’s sexuality. Indeed, during young adulthood, people are still in a phase of exploration of their sexuality and their romantic relationships (Morgan, 2013), and they may be more inclined to explore different sexual behaviors, such as sexting (Mori et al., 2020), with both stable and short-term partners (Brodie et al., 2019). Moreover, research has highlighted various individuals (Bianchi et al., 2019; Hudson & Marshall, 2016; Van Ouytsel et al., 2017, 2019) and cultural characteristics (Smahel et al., 2020) that could influence sexting behaviors. Not only, but even the socio-sanitary situation due to the COVID-19 pandemic has also affected many aspects of our lives, including our expression of sexuality, such as sexting behaviors (Bianchi et al., 2021a; Nelson et al., 2020).

Italian and Colombian cultures are quite similar in certain aspects. From a religious perspective, both are characterized by a strong influence from the catholic church, whose doctrine greatly influences sexual behavior. For example, in Italy, this results in a strong traditionalist component regarding public and private sexuality (Callahan & Loscocco, 2023).

Not only, but both countries are also still highly culturally influenced by patriarchal norms and “machismo” (Baiocco & Pistella, 2019; Baiocco et al., 2013; Kreft, 2022), and those cultural characteristics are able to influence sexual behavior, too. These two countries are similar in several aspects, but, on the other hand, are also quite different. For these reasons, the present study aimed to further explore and compare the differences in sexting behaviors between these two countries, also according to the lack of research on sexting in Colombia (Gil-Latrio, 2020). Moreover, the present study is part of a scientific agreement between the Italian Sapienza University of Rome, and the Colombian Universidad de San Buenaventura. The relevance of testing sexting behaviors in different countries allows the cross-cultural comparisons of subjects in those countries and eventually the development of increasingly universal programs aimed at education and prevention of sexting behaviors.

Consequently, this study aims to improve our knowledge about sexting in Italian and Colombian young adults before and during the COVID-19 pandemic, also exploring sex, age, and sexual orientation differences in sexting behaviors. In particular, we are interested in investigating differences in sexting behaviors such as sharing own sexts, risky sexting, non-consensual sexting, and sexting under pressure considering different cultural contexts (Italy vs. Colombia), sex, age, sexual orientation, and pandemic status.

Previous studies on cross-country comparison used a single-item measure to assess sexting behaviors that did not distinguish between different kinds of sexting (Gil-Lario et al., 2020). Instead, following the same procedure of two recent cross-cultural studies on sexting (Morelli et al., 2020, 2021a), the present study used a multi-item measure (see Appendix), i.e., the Sexting Behaviors Questionnaire (Morelli et al., 2016b), which has proved to have good psychometric properties (Morelli et al., 2016b, 2021a) and that allows assessing different dimensions of sexting: Experimental sexting (i.e., sharing own sexts), aggravated sexting (i.e., non-consensual sexting and sexting under pressure), and risky sexting (i.e., sexting during substance and alcohol use and sharing sexts with strangers met online), that previous recent cross-countries studies (Morelli et al., 2020, 2021a) identify as relevant facets of sexting behaviors with different implications for psychological and relational well-being that are worth to be investigated.

Hypothesis 1: based on the limited research studies (Gil-Lario et al., 2020), we expected that Colombian young adults were more likely to engage in sexting behaviors than Italian young adults, although with some cautions. Hypothesis 2: regarding sex, we expected that males would report a higher prevalence of sexting than females, especially in risky sexting, non-consensual sexting, and sexting under pressure. Several studies have shown that males seem to be more likely to be involved in these forms of sexting (Morelli et al., 2021a; Mori et al., 2020), probably due to their higher levels of sensation-seeking and impulsivity that bring them in being involved, in general, in more risky behaviors (Farhat et al., 2021). Exploring sex differences in sexting is also important because previous studies highlighted the presence of sexual double standards related to sexting (Ringrose et al., 2013): According to this theory, sexting may be riskier for girls than boys due to cultural pressures. Hypothesis 3: regarding age differences, we expect that older participants will report higher percentages of sharing their own sexts because sexting seems to increase with age (Morelli et al., 2021b), similar to the developmental tendency of sexual activities (Rice et al., 2014). Conversely, the negative forms of sexting will be reported more frequently by younger participants, as they show fewer considerations for the future consequences of their behaviors (Nigro et al., 2016).

Hypothesis 4: regarding sexual orientation differences, due to the sense of protection offered by online communication from prejudice and sexual stigma (Chong et al., 2015), we hypothesized that sexual minorities would report being more involved in sexting than heterosexual participants (Bianchi et al., 2019; Morelli et al., 2020; Van Ouytsel et al., 2019). Hypothesis 5: regarding the pandemic status, we expected to find higher levels of sexting behaviors in participants who took part in the study since the COVID-19 pandemic started (vs. before the COVID-19 pandemic started). Recent studies shed light on the increase in sexting behavior in young adults during the pandemic (Bianchi et al., 2021a; Lehmiller et al., 2020).

Hypothesis 6: for explorative purposes, this study will also explore the possible moderation role of the country in the relationship between each investigated variable (i.e., sex, age, sexual orientation, and pandemic status) and all different sexting behaviors (i.e., sharing own sexts, risky sexting, non-consensual sexting, and sexting under pressure, respectively).

## Method

### Participants and Procedure

Data collection in the present study was conducted from December 2019 to June 2021 both in Italy and Colombia, resulting in a total of 2931 participants (2051 from Italy and 880 from Colombia) aged 18 to 35 years old (*M*_age_ = 23.85; *SD*_age_ = 3.63; 67.6% girls). Overall, 87.1% of participants (*n* = 2553) reported being exclusively heterosexual, whereas the remaining 12.9% (*n* = 378) reported being sexual minorities (i.e., gay, lesbian, bisexual, and other non-heterosexual sexual identities; LGB +). Regarding relationship status, 52.4% of participants (*n* = 1536) reported currently having a dating partner, and 47.6% (*n* = 1395) reported currently not having one. Demographic information disaggregated by nationality is reported in Table 1.

**Table 1:** Sample characteristics by country

		Age		Sex		Sexual orientation		Dating relationship	
Countries	Sample size	Range	M(SD)	% females	% males	% Heterosexual people	% Sexual Minority people	% No	% Yes
Italy	2051	18–35	24.20 (3.47)	69.5	30.5	89.5	10.5	43.9	56.1
Colombia	880	18–35	23.06 (3.86)	63.1	36.9	81.6	18.4	56.1	43.9

An English version of the questionnaire was shared between Italian and Colombian researchers. Therefore, before starting data collection, each country worked on a language adaptation of the survey: Native speaker researchers translated and back-translated the survey. Then, the two final versions (Italian and Colombian) were compared. This process did not highlight any specific differences between the Italian and Colombian versions of the survey. The study was conducted according to the guidelines of the Declaration of Helsinki and approved by the Ethics Committee of the Sapienza University of Rome.

Participants were invited to take part in a study on relationships in the online context. Participants were given no further specific information on the investigated variables to not affect their answers. All recruited participants completed an online survey hosted by the Unipark platform. They were recruited through a snowball sampling procedure: Initial contacts were through university mailing lists, contacts with LGBT + associations, and contacts through the main social networks, in both countries. Then, they were asked to share the survey link among their contacts and social networks. The anonymity was guaranteed, and participants gave their informed consent by clicking on “yes, I accept to participate” on the first page of the survey. Only the questionnaires filled out were considered valid among all the participants reached. Thus, the response rate was 93%. The duration of the survey was 20–25 min.

### Measures

#### Socio-demographic Variables

Participants reported their age, sex (0 = male; 1 = female) and dating relationship status (0 = not having a partner; 1 = currently having a partner). Participants indicated their sexual orientation using 1 for exclusively heterosexual, 2 for bisexual, 3 for gay, 4 for lesbian, and 5 for other non-heterosexual sexual identities such as pansexual, asexual, or demisexual. Following the procedure used in previous studies (e.g., Pistella et al., 2019), respondents were categorized as exclusively heterosexual (who answered 1) and sexual minority youth (who answered from 2 to 5).

#### Sexting Behaviors

Sexting was defined as the sending and publicly posting sexually suggestive and provocative text messages/photos/videos (i.e., sexts) via the Internet or smartphone. Following the same procedure used by ), 18 items from the Sexting Behaviors Questionnaire (SBQ; Morelli et al., 2016b) were administered to assess the frequency of four sexting behaviors during the last year. Each item was rated on a 5-point Likert scale from 1 = *Never* to 5 = *Always* or *almost daily*. Experimental sexting, sending and publicly posting own sexts, was measured with four items (sample item is “How often have you publicly post provocative or sexually suggestive videos about yourself?”; Cronbach’s alpha of 0.66). Risky sexting, which is engaging in sexting during substance and alcohol use and sharing sexts with strangers met online, was evaluated with four items (a sample item is “Sometimes I sext when I drink alcohol”; Cronbach’s alpha of 0.63).

As regards aggravated sexting, it was assessed both the perpetration dimension, that is the non-consensual sexting, consisting of privately sending and publicly posting sexts of someone else (i.e., a partner or an acquaintance) without his/her consent (8 items; a sample item is “How often have you privately sent sexually suggestive or provocative photos about your partner without his/her consent?”; Cronbach’s alpha was 0.88), and the victimization dimensions, that is sharing sexts under the pressure of partner or friends (2 items; a sample item is “Sometimes I sext because my partner forced me”; Cronbach’s alpha was 0.65).

### Data Analysis

All investigated sexting behaviors exhibited a strongly polarized non-normal distribution, with participants reporting never to have been engaged in sexting ranging from about 43 to 97%, depending on the different dimensions. Therefore, variables were coded as non-sexters (participants who always answered 1 to all items of the SBQ, which means that they have never been engaged in sexting) and sexters (participants who have reported more than 1 on at least one item, which means that they have been engaged in sexting at least once). Moreover, a variable called “pandemic status” was created ad hoc: Participants recruited before the COVID-19 pandemic started (i.e., before the first lockdown in March 2020; *n* = 1372, 46.8%) were coded as 0, and participants recruited since the pandemic began (i.e., collected from March 2020 onwards; *n* = 1559, 53.2%) were coded as 1 to investigate differences in sexting behaviors due to the diffusion of the pandemic and the restrictive measures that limited social interactions (Bianchi et al., 2021a).

The prevalence of different sexting behaviors was calculated. A series of chi-square analyses were conducted to investigate differences between Colombia and Italy in each kind of sexting (hypothesis 1). Finally, four logistic regression analyses were run to analyze (hypothesis 2) the effect of sex (0 = male; 1 = female), (hypothesis 3) age (years), (hypothesis 4) sexual orientation (0 = exclusively heterosexual; 1 = sexual minority status), (hypothesis 5) pandemic status (0 = before pandemic; 1 = since the pandemic started), country-level (0 = Colombia; 1 = Italy), and (hypothesis 6) the effect of interaction terms between each of these variables and the country (sex*country; age*country; sexual identity*country; pandemic status*country) on the different sexting behaviors (i.e., sharing own sexts, risky sexting, non-consensual sexting, and sexting under pressure).

When analyses were run for non-consensual sexting and sexting under pressure, the logistic regressions were conducted only on the subsample of participants who reported currently having a dating partner (*n* = 1536; *M*_age_ = 24.39; *SD*_age_ = 3.66; age range = 18–35; 71.7% girls; 89.1% exclusively heterosexual), since these two dimensions of aggravated sexting comprised items about sexting behaviors with a dating partner. All analyses were performed using the software SPSS 25.

## Results

### Prevalence of Sexting Behaviors

Regarding the total sample, 57.7% (*n* = 1691) of participants reported having shared their own sext at least once, and 38% (*n* = 1113) had engaged in risky sexting at least once. In the subsample of participants who reported currently having a dating relationship, 9.8% (*n* = 150) reported having engaged in non-consensual sexting at least once, and 4% (*n* = 61) had been pressured to sext at least once.

To assess the first hypothesis, a series of chi-square analyses were run to explore country differences (Colombia versus Italy) in percentage frequencies of each sexting behavior. Country differences emerged in risky sexting, χ^2^ (1) = 13.45, *p* < .001, and in non-consensual sexting, χ^2^ (1) = 8.03, *p* = .005. Specifically, Italian youth reported being more engaged in risky sexting than Colombian youth, while Colombians reported being more engaged in non-consensual sexting than Italians. No differences between Italy and Colombia were found in sharing own sexts, χ^2^ (1) = 0.35, *p* = .85, and in sexting under pressure, χ^2^ (1) = 0.01, *p* = .92. Table 2 reports the percentages and frequencies of sexters and non-sexters for males and females in each country.

**Table 2:** Percentage and frequencies of sexters and non-sexters for males and females by country

	Country			
	Colombia		Italy	
	Males % (*n*)	Females % (*n*)	Males % (*n*)	Females % (*n)*
Sharing own sext				
Non-sexters	41.5 (135)	42.3 (235)	45.4 (284)	41.1 (586)
Sexters	58.5 (190)	57.7 (320)	54.6 (341)	58.9 (840)
Risky sexting				
Non-sexters	61.2 (199)	70.5 (391)	56.4 (353)	61.4 (875)
Sexters	38.8 (126)	29.5 (164)	43.5 (272)	38.6 (551)
Non-consensual sexting				
Non-sexters	79.6 (109)	90.4 (225)	83.5 (248)	94.3 (804)
Sexters	20.4 (28)	9.6 (24)	16.5 (49)	5.7 (49)
Sexting under pressure				
Non-sexters	96.4 (132)	96 (239)	93.6 (278)	96.8 (826)
Sexters	3.6 (5)	4.0 (10)	6.4 (19)	3.2 (27)

### Logistic Regression Analyses

To assess hypotheses 2, 3, 4, 5, and 6, a series of logistic regressions were run to investigate each research question on the dependent variable. In the first logistic regression analysis were tested the effects of sex, age, sexual identity, pandemic status, country level, and the effect of interaction terms between each of these variables and the country (sex*country; age*country; sexual identity*country; pandemic status*country) on sharing own sexts. The model accounted for 3.3% of the variance, Nagelkerke’s *R*-squared = 0.033, *χ*^2^(9) = 72.92, *p* < .001. Sharing own sexts was positively related to sexual orientation, *b* = 1.11, *p* < .001, and to pandemic status, *b* = 0.37, *p* = .02. Specifically, sexual minority people reported 3 times more sharing their own sexts than exclusively heterosexual counterparts, OR = 3.02. Moreover, participants sent their own sexts 1.5 times more during the pandemic than in the pre-pandemic era, OR = 1.45. No significant effects of sex, age, country, and no significant interaction effects were found.

The second logistic regression analysis tested the effects of sex, age, sexual identity, pandemic status, country level, and the effect of interaction terms between each of these variables and the country on risky sexting. The model accounted for 4.7% of the variance, Nagelkerke’s *R*-squared = 0.047, *χ*^2^(9) = 103.47, *p* < .001. Risky sexting was negatively related to sex, *b* =  − 0.38, *p* = .012, and positively related to sexual orientation, *b* = 1.19, *p* < .001. Specifically, females involved less than males in risky sexting by approximately 31.6%, OR = 0.684, and sexual minority people reported 3.2 times more risky sexting than exclusively heterosexual counterparts, OR = 3.29. No significant effects of age, pandemic status, country, and no significant interaction effects were found.

After that, on the subsample of participants reporting to have a partner currently, a logistic regression analysis was run to investigate the effects of sex, age, sexual orientation, pandemic status, country level, and the effect of interaction terms between each of these variables and the country on non-consensual sexting. The model accounted for 7.6% of the variance, Nagelkerke’s *R*-squared = 0.076, *χ*^2^(9) = 56.44, *p* < .001. Non-consensual sexting was negatively related to sex, *b* =  − 0.95, *p* = .002, and to pandemic status, *b* =  − 0.80, *p* = .02, and positively related to sexual identity, *b* = 0.81, *p* = .03. Specifically, females involved less than males in non-consensual sexting by approximately 61.5%, OR = 0.38, and sexual minority youth reported 2.2 times more non-consensual sexting than exclusively heterosexual youth, OR = 2.26. Moreover, during the pandemic, participants were less involved in non-consensual sexting than in the pre-pandemic of 55%, OR = 0.45. No significant effects of age, country, and no significant interaction effects were found.

Finally, a logistic regression analysis tested the effects of sex, age, sexual identity, pandemic status, country level, and the effect of interaction terms between these variables and the country on sexting under pressure. The model was not significant, accounting for 2.6% of the variance, Nagelkerke’s *R*-squared = 0.026, *χ*^2^(9) = 11.21, *p* = .26. Nevertheless, sexting under pressure was positively related only to sexual orientation, *b* = 1.35, *p* = .01. Thus, sexual minority people reported 3.8 times more sexting under pressure than exclusively heterosexual youth, OR = 3.87. See Table 3 for detailed statistics and odds ratios of each logistic regression.

**Table 3:** Logistic regression analyses: sexting behaviors

	Sexting behaviors															
	Own sexting				Risky sexting				Non-consensual sexting				Sexting under pressure			
Predictors	*B*	*SE*	*OR*	*95% CI*	*B*	*SE*	*OR*	*95% CI*	*B*	*SE*	*OR*	*95% CI*	*B*	*SE*	*OR*	*95% CI*
Sex	0.03	0.15	1.02	[0.77, 1.36]	− 0.38*	0.15	0.68	[0.51, 0.92]	− 0.95**	0.31	0.38	[0.21, 0.71]	0.11	0.57	1.12	[0.37, 3.41]
Age	− 0.03	0.02	0.97	[0.93, 1.00]	− 0.002	0.02	0.99	[0.96, 1.04]	− 0.03	0.04	0.97	[0.89, 1.05]	0.01	0.07	1.01	[0.89, 1.15]
Sexual orientation	1.11***	0.2	3.02	[2.02, 4.51]	1.19***	0.18	3.29	[2.31, 4.68]	0.81*	0.37	2.26	[1.10, 4.65]	1.35*	0.55	3.87	[1.31, 11.42]
Pandemic	0.37*	0.17	1.45	[1.05, 2.01]	− 0.05	0.18	0.95	[0.67, 1.35]	− 0.80*	0.33	0.45	[0.23, 0.86]	− 0.13	0.62	0.88	[0.26, 2.96]
Country	0.3	0.2	1.34	[0.91, 1.99]	0.79	0.61	2.21	[0.67, 7.26]	− 1.91	1.29	0.15	[0.01, 1.87]	1.72	2,14	5.56	[0.08, 372.36]
BS*C	0.13	0.18	1.14	[0.80, 1.60]	0.15	0.18	1.17	[0.82, 1.66]	− 0.21	0.38	0.81	[0.39, 1.70]	− 0.87	0.65	0.42	[0.12, 1.49]
A*C	0.01	0.02	1.01	[0.96, 1.05]	− 0.02	0.02	0.98	[0.94, 1.03]	0.05	0.05	1.05	[0.96, 1.16]	− 0.04	0.08	0.96	[0.82, 1.13]
SI*C	− 0.41	0.26	0.66	[0.39, 1.09]	− 0.40	0.23	0.67	[0.42, 1.05]	− 0.52	0.51	0.59	[0.22, 1.64]	− 1.37	0.77	0.25	[0.06, 1.16]
P*C	− 0.30	0.19	0.74	[0.51, 1.08]	0.05	0.2	1.05	[0.71, 1.55]	0.51	0.4	1.66	[0.76, 3.65]	0.19	0.69	1.21	[0.31, 4.69]

Sex was coded as 0 = male and 1 = female. Sexual identity was coded as 0 = exclusively heterosexual and 1 = lesbian, gay, bisexual; Pandemic was coded as 0 = before the COVID-19 pandemic and 1 = since the pandemic started. Country was coded as 0 = Colombia and 1 = Italy. BS*C = sex*country; A*C = age*country; SI*C = sexual identity*country; P*C = pandemic *country. Logistic regression coefficients are reported

*OR* odds ratio, *CI* 95% confidence interval

*p < .05; **p < .01; ***p < .001

## Discussions

The present study investigates differences in sexting behaviors (e.g., sharing of own sext, risky sexting, non-consensual sexting, and sexting under pressure) between Italian and Colombian young adults before and during the COVID-19 pandemic period. One strength of this study was the examination of sexting through a validated multi-item sexting measure, showing good psychometric properties (Morelli et al., 2016b). Indeed, the results from a recent review (Barrense-Dias et al., 2017) have highlighted the difficulty in comparing data of individual studies conducted in various countries because each study measured sexting differently. Again, most studies have used a single-item measure to assess sexting behaviors without distinguishing different kinds of sexting (Gil-Lario et al., 2020).

Hypothesis 1 is partly confirmed: Italian youths were more engaged in risky sexting than Colombians, while Colombian young adults were more engaged in non-consensual sexting than Italians. Within sexting behaviors considered in this study, the non-consensual represents the more violent form of sexting, belonging to the aggravated sexting. This result is in line with previous research: Gil-Lario and colleagues (2020) found a higher level of sexting behavior in Colombian people compared to Spain participants. The authors suggested that this difference could be due to more gender inequalities in Colombia than in Spain, related to involvement in risky online sexual behaviors. Future studies should address the stability and the meaning of this finding over time.

Hypothesis 2 is partly confirmed. According to previous studies, males reported higher levels of non-consensual and risky sexting than females (Morelli et al., 2020): Males typically report higher rates of general risky behaviors (Weden & Zabin, 2005) and sexual risky behaviors (Eaton et al., 2008) than females. In particular, males’ attitude to underemphasize empathy and self-regulation and emphasize self-assertion may bring them a higher risk of externalizing problems (Leadbeater et al., 1999), reflecting more involvement in risky behaviors, such as risky sexting and non-consensual sexting.

Results seem to suggest that males are more involved in aggravated sexting; this could implicate that females could be more victims of these behaviors, as suggested by the sexual double standard theory (Ringrose et al., 2013). Not only, but stereotypes of masculinity and femininity that pervade society could also contribute to these disparities. According to the masculine gender role, men should always be willing to have sexual intercourse, which can influence the higher rates of their sexting behaviors (Ramiro-Sánchez et al., 2018).

Moreover, the feminine gender roles perpetuate the ideals associated with virginity, innocence, sexual passivity, and sexual terror which can influence the lower rates of their sexting behaviors (Barjola, 2018; Morero Beltrán & Camps Calvet, 2019; Sáncehz Ramos, 2018). Finally, some studies suggest a gendered use of technology (Vergés Bosh et al., 2021). In particular, it would appear that males are more involved in technology use than females, which could explain the higher prevalence of sexting among males. Hypothesis 3 is confirmed. The findings of the present study are in line with previous studies (Morelli et al., 2020, 2021a): Young adults are more likely to be involved in sharing their own sexts, followed by risky sexting, and to a lesser extent, albeit clinically relevant, in non-consensual and sexting under pressure. Hypothesis 4 is confirmed: results regarding sexual orientation differences have shown that non-heterosexual sexual orientation predicted higher levels of each sexting behavior (sharing own sexting, risky sexting, non-consensual sexting, and sexting under pressure). Previous studies (Bianchi et al., 2019; Morelli et al., 2020; Van Ouytsel et al., 2019) found that LGB + young adults were more involved in sexting behavior than heterosexual people. LGB + people are exposed to prejudice and sexual stigma (Meyer, 2003), and the social media and “virtual” environment could be a protective factor against minority stressors allowing them to be “less exposed” and bringing them to a higher involvement in social and virtual behaviors. Indeed, research has shown that social media can facilitate communication and relationships among sexual minority people and improve their social-emotional and psychological well-being (Chong et al., 2015). Our results show that a higher prevalence of LGB + people is involved in risky sexting (3.2 times more than heterosexual counterparts). These findings align with other studies which suggest that LGB + people are more at risk for substance consumption (Goldbach et al., 2017).

Hypothesis 5 was partially confirmed: the COVID-19 pandemic brought higher levels of sharing experimental sexting and lower levels of non-consensual sexting, considering sexting as a virtual sex a good option during and after lockdown (Bianchi et al., 2021a). The fear of contagion and home confinement could have motivated youth to engage in experimental sexting. For instance, couples that were not cohabiting and were forced into a long-distance relationship (Wijayanti, 2021) could have felt that sexting was the best alternative to maintain intimacy and sexual desire (Bianchi et al., 2021a). Even for people who used to be involved in many casual sexual relationships, which were forbidden during the pandemic (Wignall et al., 2021), sexting and other virtual sexual activities might have been the easiest and safest way to satisfy their sexual desires.

Moreover, it seems that the COVID-19 pandemic was related to lower levels of non-consensual sexting. During this period, young adults have considered non-consensual sexting a helpful behavior to cope with stress (Bianchi et al., 2021a) and to explore and keep their sexuality active despite restrictions rather than a way to perpetrate violence against their partners and other people. Thus, findings seem to suggest the importance of conducting studies investigating how motivations for involving in sexting have changed since the pandemic started.

## Limits and Future Directions

The present study involved some significant limitations, which should be addressed in future research. The first is the use of self-report measures: there could be a possible social desirability effect, especially in participants with a less liberal or more traditional culture of sexuality. However, online data collection may have limited this effect by ensuring a greater sense of privacy. Future research could benefit from using measures to assess individuals’ implicit beliefs or using semistructured interviews to investigate sexting behaviors. A second limit could lie in the generalizability of the results. Although participants have been recruited across two countries, results can only be interpreted in Italy and Colombia. Cross-cultural studies are essential to understand how certain behaviors work across cultures and how each country’s characteristics influence them. From this perspective, replicating studies involving different countries is critical. The third limit resides in the lack of data regarding the participants’ culture. These data could be helpful to understand better the cultural dynamics underlying the differences in sexting behavior revealed by the results. Future studies could consider dimensions such as personal and cultural openness regarding sex.

A fourth limit can reside in considering a unique group of sexual minority participants rather than differentiating them by gender and/or sexual orientation. Future studies could try to improve the specificity and representativity of sexual minorities by considering the different groups that are part of it. The fifth limit could be the difference between the Italian and Colombian sample size and sample composition. There were many more Italian respondents than Colombian respondents, and in the latter, the percentage of sexual minority people was higher. This could influence the representativeness and generalizability of the results in the different contexts considered, as well as the results of their comparisons. Future studies might seek to recruit more homogeneous samples. However, the investigated variables were added as predictors in the regression analyses. So, their eventual influences were accounted for and taken into consideration.

A sixth limit can reside in the low Nagelkerke’s *R*-squared (even if significant), as good fit values are considered between 0.02 and 0.03 (López-Roldan & Fachelli, 2015). The explained variance is rather small, so it is important considering that the findings did not explain a high percentage of the variance. However, the explained percentage of variance is statistically significant, and this indicates that there is an effect of predictors on the dependent variable that cannot be ignored and it is not simply due to chance.

Moreover, as regards sexting under pressure, the percentage frequencies are low, and this is reflected in the fact that the percentage of sexters is less than 10% of the total sample. This implies that one must be cautious in interpreting the results. Future studies could consider within their study other variables, both individual and cultural, that may be able to influence sexting behaviors. Finally, future studies could benefit from a more in-depth analysis of sexting behaviors concerning the content sent and the implicit and explicit meanings people give to such behaviors. Sexting behaviors are relatively recent, as is the research on them, so many dimensions and characteristics still require further investigation.

### Policy Implications

Despite these limitations, this cross-cultural study brings implications for educational and prevention programs. During young adulthood, a developmental phase in which individuals explore their sexuality and the adequacy of their body image (Bianchi et al., 2021b; Morelli et al., 2017b), the consensual exchange of sexts can help people address their developmental tasks and needs related to the exploration and construction of their sexuality and identity (Bianchi et al., 2019; Kosenko et al., 2017; Morelli et al., 2021a). A better understanding of sexual activities such as sexting during all developmental phases and the differences in sexting behavior between different cultures can help implement programs to instill greater awareness in young people about sexting and sexual behaviors faced during this developmental stage.

Although our study was conducted on a sample of young adults, it would be essential to conduct prevention programs starting from adolescence to sensitize teens to a more conscious and safe use of sexting, the Internet, and social networks. These programs should increase teens’ awareness of the risks and consequences of sexting, which often leads them to get involved in poorly adaptive relationships. Since sexting behaviors also play an essential role within couple relationships and can become a tool for acting violently against the partner, prevention and intervention programs should also focus on promoting positive relational patterns among youth, emphasizing respect for others and privacy. Indeed, young people are unfamiliar with intimate relationships, so some kinds of aggression are interpreted as rudimentary means for expressing intimacy and interest and resolving conflicts, exposing them to coercive and violent dynamics (Wekerle & Wolfe, 1999).

Another recommendation concerns awareness-raising in the sphere of health services. Mental health and clinical psychology centers should consider how sexting behavior, particularly non-consensual sexting, might impact young adults’ well-being and ability to maintain respectful and intimate relationships with other people. The present findings suggest that mental health agencies should: (a) recognize the need to adequately and appropriately prepare psychologists and counselors to consider the relevance of sexting behaviors, ensuring respectful listening, awareness of one’s prejudices, and adequate knowledge of sexting behaviors; (b) address the different meanings and motivations that the sexting behaviors may have for young adult people and in particular for sexual minority people; and (c) provide specific suggestions for work with clients with at online risk behaviors, due to the spread of technologies and Internet availability during the COVID-19 pandemic, modifying our way of communicating with others, including sexual communication.

## Data Availability

Data are available under request to the first author.

## Appendix

### Items used to investigate sexting behaviors

#### Own sexting

- In the last 12 months, how often have you privately sent provocative or sexually suggestive photos about yourself?
- In the last 12 months, how often have you publicly posted provocative or sexually suggestive photos about yourself?
- In the last 12 months, how often have you privately sent provocative or sexually suggestive videos about yourself?
- In the last 12 months, how often have you publicly posted provocative or sexually suggestive videos about yourself?

#### Risky sexting

- Sometimes I sext when I drink alcohol.
- Sometimes I sext when I am smoking marijuana.
- Sometimes I sext during substance use.
- Sometimes I sext with strangers met online.

#### Non-consensual sexting

- How often have you privately sent sexually suggestive or provocative photos about someone you know without his/her consent?
- How often have you publicly posted sexually suggestive or provocative photos about your partner without his/her consent?
- How often have you publicly posted sexually suggestive or provocative photos about someone you know without his/her consent?
- How often have you privately sent sexually suggestive or provocative videos about your partner without his/her consent?
- How often have you privately sent sexually suggestive or provocative videos about someone you know without his/her consent?
- How often have you publicly posted sexually suggestive or provocative videos about your partner without his/her consent?
- How often have you publicly posted sexually suggestive or provocative videos about someone you know without his/her consent?

#### Sexting under pressure

- Sometimes I sext because my partner forced me.
- Sometimes I sext because my friends forced me.

## References

[CR1] Albury K, Crawford K (2012). Sexting, consent and young people’s ethics: Beyond Megan’s story. Continuum.

[CR2] Armour, C., McGlinchey, E., Butter, S., McAloney-Kocaman, K., & McPherson, K. E. (2021). The COVID-19 psychological well-being study: Understanding the longitudinal psychosocial impact of the COVID-19 pandemic in the UK; A methodological overview paper. *Journal of**Psychopathology and Behavioral Assessment, 43*(1), 174–190. 10.31234/osf.io/9p4tv 10.1007/s10862-020-09841-4 PMC7641483 33169046

[CR3] Babore, A., Morelli, M., & Trumello, C. (2021). Italian adolescents’ adjustment before and during the coronavirus disease, 2019 A comparison between mothers’ and adolescents’ perception. *British Journal of Clinical Psychology,**61(*2), 281–286. 10.1111/bjc.12334 10.1111/bjc.12334 PMC8646383 34585749

[CR4] Babore, A., Trumello, C., Lombardi, L., Candelori, C., Chirumbolo, A., Cattelino, E., & Morelli, M. (2021b). Mothers’ and children’s mental health during the COVID-19 pandemic lockdown: The mediating role of parenting stress. *Child Psychiatry & Human Development,* 1–13. 10.1007/s10578-021-01230-6 10.1007/s10578-021-01230-6 PMC8379586 34417927

[CR5] Baiocco R, Nardelli N, Pezzuti L, Lingiardi V (2013). Attitudes of Italian heterosexual older adults towards lesbian and gay parenting. Sexuality Research and Social Policy.

[CR6] Baiocco R, Pistella J (2019). “Be as you are” clinical research center at the Sapienza University of Rome. Journal of Gay & Lesbian Mental Health.

[CR7] Ballester-Arnal R, Nebot-Garcia JE, Ruiz-Palomino E, Giménez-García C, Gil-Llario MD (2020). “INSIDE” project on sexual health in Spain: Sexual life during the lockdown caused by COVID-19. Sexuality Research and Social Policy.

[CR8] Barjola N (2018). Microfísica sexista del poder. El caso Alcàsser y la construcción del terror sexual.

[CR9] Barrense-Dias Y, Berchtold A, Surís JC, Akre C (2017). Sexting and the definition issue. Journal of Adolescent Health.

[CR10] Benotsch EG, Snipes DJ, Martin AM, Bull SS (2013). Sexting, substance use, and sexual risk behavior in young adults. Journal of Adolescent Health.

[CR11] Bianchi, D., Baiocco, R., Lonigro, A., Pompili, S., Zammuto, M., Di Tata, D., & Laghi, F. (2021a). Love in quarantine: Sexting, stress, and coping during the COVID-19 lockdown. *Sexuality Research and Social Policy*, 1–14. 10.1007/s13178-021-00645-z 10.1007/s13178-021-00645-z PMC8458047 34580599

[CR12] Bianchi D, Morelli M, Baiocco R, Chirumbolo A (2017). Sexting as the mirror on the wall: Body-esteem attribution, media models, and objectified-body consciousness. Journal of Adolescence.

[CR13] Bianchi D, Morelli M, Baiocco R, Cattelino E, Laghi F, Chirumbolo A (2019). Family functioning patterns predict teenage girls’ sexting. International Journal of Behavioral Development.

[CR14] Bianchi D, Morelli M, Nappa MR, Baiocco R, Chirumbolo A (2021). A bad romance: Sexting motivations and teen dating violence. Journal of Interpersonal Violence.

[CR15] Bianchi D, Morelli M, Baiocco R, Cattelino E, Chirumbolo A (2021). Patterns of love and sexting in teen dating relationships: The moderating role of conflicts. New Directions for Child and Adolescent Development.

[CR16] Brodie ZP, Wilson C, Scott GG (2019). Sexual intercourse: Considering social–cognitive predictors and subsequent outcomes of sexting behavior in adulthood. Archives of Sexual Behavior.

[CR17] Callahan I, Loscocco K (2023). The prevalence and persistence of homophobia in Italy. Journal of Homosexuality.

[CR18] Chalfen R (2009). ‘It’s only a picture’: Sexting, ‘smutty’ snapshots and felony charges. Visual Studies.

[CR19] Chong ESK, Zhang Y, Mak WWS, Pang IHY (2015). Social media as social capital of LGB individuals in Hong Kong: Its relations with group membership, stigma, and mental well-being. American Journal of Community Psychology.

[CR20] Currin JM, Hubach RD (2019). Motivations for nonuniversitybased adults who sext their relationship partners. Journal of Sex & Marital Therapy.

[CR21] Döring, N. (2014). Consensual sexting among adolescents: Risk prevention through abstinence education or safer sexting. *Cyberpsychology: Journal of Psychosocial Research on Cyberspace, 8*(1), 9. 10.5817/cp2014-1-9

[CR22] Döring N (2020). How is the COVID-19 pandemic affecting our sexualities? An overview of the current media narratives and research hypotheses. Archives of Sexual Behavior.

[CR23] Drouin M, Tobin E (2014). Unwanted but consensual sexting among young adults: Relations with attachment and sexual motivations. Computers in Human Behavior.

[CR24] Drouin M, Coupe M, Temple JR (2017). Is sexting good for your relationship? It depends. Computers in Human Behavior.

[CR25] Eaton, D. K., Kann, L., Kinchen, S., Shanklin, S., Ross, J., Hawkins, J., & Centers for Disease Control and Prevention (CDC). (2008). Youth risk behavior surveillance—United States, 2007. *Morbidity and Mortality Weekly Report, 57*(4), 1–131. 18528314

[CR26] Eisenman R, Dantzker ML (2006). Gender and ethnic differences in sexual attitudes at a Hispanic-serving university. The Journal of General Psychology.

[CR27] Eleuteri S, Terzitta G (2021). Sexuality during the COVID-19 pandemic: The importance of Internet. Sexologies.

[CR28] Farhat LC, Wampler J, Steinberg MA, Krishnan-Sarin S, Hoff RA, Potenza MN (2021). Excitement-seeking gambling in adolescents: Health correlates and gambling-related attitudes and behaviors. Journal of Gambling Studies.

[CR29] Gabster, A., Erausquin, J. T., Michielsen, K., Mayaud, P., Pascale, J. M., Escale, C. P., & Tucker, J. D. (2021). How did COVID-19 measures impact sexual behaviour and access to STI&HIV services in Panama? Results from a national cross-sectional online survey. *Sexually Transmitted Infections*. 10.1136/sextrans-2021-054985 10.1136/sextrans-2021-054985 34400575

[CR30] Gámez-Guadix M, Mateos-Pérez E (2019). Longitudinal and reciprocal relationships between sexting, online sexual solicitations, and cyberbullying among minors. Computers in Human Behavior.

[CR31] Gassó AM, Klettke B, Agustina JR, Montiel I (2019). Sexting, mental health, and victimization among adolescents: A literature review. International Journal of Environmental Research and Public Health.

[CR32] Gassó AM, Mueller-Johnson K, Agustina JR, Gómez-Durán E (2021). Mental health correlates of sexting coercion perpetration and victimisation in university students by gender. Journal of Sexual Aggression.

[CR33] Gil-Llario MD, Morell-Mengual V, Jiménez-Martínez MC, Iglesias-Campos P, Gil-Julia B, Ballester-Arnal R (2020). Culture as an influence on sexting attitudes and behaviors: A differential analysis comparing adolescents from Spain and Colombia. International Journal of Intercultural Relations.

[CR34] Goldbach JT, Mereish EH, Burgess C (2017). Sexual orientation disparities in the use of emerging drugs. Substance Use & Misuse.

[CR35] Gonzalez ER, Argel MM, Guzman MA, Otalvaro AR, Reyes DD (2021). Sexting in young university of the Colombian Caribbean, a comparative study between male and female. European Psychiatry.

[CR36] Hudson HK, Marshall SA (2016). Sexty southerners: Sexting content and behaviors among selected southern undergraduates. Health Education.

[CR37] International Society for the Study of Women's Sexual Health. (2020). Position statement on sexual activity and COVID-19. Retrieved May 2022, from https://www.isswsh.org/images/COVID_Position_Statement_5-08-20_-_revised.pdf

[CR38] Kosenko K, Luurs G, Binder AR (2017). Sexting and sexual behavior, 2011–2015: A critical review and meta-analysis of a growing literature. Journal of Computer-Mediated Communication.

[CR39] Kreft AK (2022). “This Patriarchal, Machista and Unequal Culture of Ours”: Obstacles to Confronting Conflict-Related Sexual Violence.

[CR40] Leadbeater BJ, Kuperminc GP, Blatt SJ, Hertzog C (1999). A multivariate model of gender differences in adolescents' internalizing and externalizing problems. Developmental Psychology.

[CR41] Lehmiller JJ, Garcia JR, Gesselman AN, Mark KP (2020). Less sex, but more sexual diversity: Changes in sexual behavior during the COVID-19 coronavirus pandemic. Leisure Sciences.

[CR42] López-Roldan, P., & Fachelli, S. (2015). *Metodología de la Investigación Social Cuantitativa* [Methodology in social quantitative research]. Available online: http://ddd.uab.cat/record/129382

[CR43] Madigan S, Ly A, Rash CL, Van Ouytsel J, Temple JR (2018). Prevalence of multiple forms of sexting behavior among youth: A systematic review and meta-analysis. JAMA Pediatrics.

[CR44] Meyer IH (2003). Prejudice, social stress, and mental health in lesbian, gay and bisexual populations: Conceptual issues and research evidence. Psychological Bulletin.

[CR45] Morelli M, Bianchi D, Baiocco R, Pezzuti L, Chirumbolo A (2016). Not-allowed sharing of sexts and dating violence from the perpetrator’s perspective: The moderation role of sexism. Computers in Human Behavior.

[CR46] Morelli M, Bianchi D, Baiocco R, Pezzuti L, Chirumbolo A (2016). Sexting, psychological distress and dating violence among adolescents and young adults. Psicothema.

[CR47] Morelli M, Bianchi D, Baiocco R, Pezzuti L, Chirumbolo A (2017). Sexting behaviors and cyber pornography addiction among adolescents: The moderating role of alcohol consumption. Sexuality Research and Social Policy.

[CR48] Morelli M, Bianchi D, Cattelino E, Nappa MR, Baiocco R, Chirumbolo A (2017). Quando il sexting diventa una forma di violenza? Motivazioni al sexting e dating violence nei giovani adulti. Maltrattamento e Abuso All’infanzia.

[CR49] Morelli, M., Chirumbolo, A., Bianchi, D., Baiocco, R., Cattelino, E., Laghi, F., & Drouin, M. (2020). The role of HEXACO personality traits in different kinds of sexting: A cross-cultural study in 10 countries. *Computers in Human Behavior*, *113*, 106502.

[CR50] Morelli, M., Cattelino, E., Trumello, C., Longobardi, E., & Baiocco, R. (2021b). Parents' psychological factors promoting children's mental health and emotional regulation during the COVID-19 lockdown. *Abuso e Maltrattamento all’Infanzia*, 47–63. 10.3280/mal2021a001004

[CR51] Morelli, M., Urbini, F., Bianchi, D., Baiocco, R., Cattelino, E., Laghi, F., & Chirumbolo, A. (2021a). The relationship between dark triad personality traits and sexting behaviors among adolescents and young adults across 11 countries. *International Journal Of Environmental Research and Public Health*, *18*(5), 2526. 10.3390/ijerph18052526 10.3390/ijerph18052526 PMC7967332 33806314

[CR52] Morero Beltrán A, Camps Calvet C (2019). La respuesta del movimiento feminista a la violencia sexual en el espacio público. La agresión sexual múltiple en las fiestas de San Fermín de 2016 como punto de inflexión. Anuari Del Conflicte Social.

[CR53] Morgan EM (2013). Contemporary issues in sexual orientation and identity development in emerging adulthood. Emerging Adulthood.

[CR54] Mori C, Cooke JE, Temple JR, Ly A, Lu Y, Anderson N, Madigan S (2020). The prevalence of sexting behaviors among emerging adults: A meta-analysis. Archives of Sexual Behavior.

[CR55] Mori C, Temple JR, Browne D, Madigan S (2019). Association of sexting with sexual behaviors and mental health among adolescents: A systematic review and meta-analysis. JAMA Pediatrics.

[CR56] Nelson KM, Gordon AR, John SA, Stout CD, Macapagal K (2020). “Physical sex is over for now”: Impact of COVID-19 on the well-being and sexual health of adolescent sexual minority males in the US. Journal of Adolescent Health.

[CR57] Nigro G, Cosenza M, Ciccarelli M, Joireman J (2016). An Italian translation and validation of the Consideration of Future Consequences-14 Scale. Personality and Individual Differences.

[CR58] Parker TS, Blackburn KM, Perry MS, Hawks JM (2013). Sexting as an intervention: Relationship satisfaction and motivation considerations. The American Journal of Family Therapy.

[CR59] Pistella J, Ioverno S, Russell ST (2019). The role of peer victimization, sexual identity, and gender on unhealthy weight control behaviors in a representative sample of Texas youth. International Journal of Eating Disorders.

[CR60] Pistella, J., Isolani, S., Ioverno, S., Laghi, F., & Baiocco, R. (2022). Psychophysical Impact of COVID-19 Pandemic and Same-Sex Couples’ Conflict: The Mediating Effect of Internalized Sexual Stigma. *Frontiers in Psychology, 13,* 860260. 10.3389/fpsyg.2022.860260 10.3389/fpsyg.2022.860260 PMC8965871 35369158

[CR61] Ramiro-Sánchez T, Ramiro MT, Bermúdez MP, Buela-Casal G (2018). Sexism and sexual risk behavior in adolescents: Gender differences. International Journal of Clinical and Health Psychology.

[CR62] Ramos, M. S. (2018). La reapropiación feminista del relato del terror sexual: El Caso Alcàsser. *IC Revista Científica de Información y Comunicación, 15*.

[CR63] Rice E, Gibbs J, Winetrobe H, Rhoades H, Plant A, Montoya J, Kordic T (2014). Sexting and sexual behavior among middle school students. Pediatrics.

[CR64] Ringrose J, Harvey L, Gill R, Livingstone S (2013). Teen girls, sexual double standards and ‘sexting’: Gendered value in digital image exchange. Feminist Theory.

[CR65] Smahel, D., Machackova, H., Mascheroni, G., Dedkova, L., Staksrud, E., Ólafsson, K., & Hasebrink, U. (2020). EU Kids Online 2020: Survey results from 19 countries. 10.21953/lse.47fdeqj01ofo

[CR66] Temple JR (2015). A primer on teen sexting. JAACAP Connect.

[CR67] Trumello, C., Bramanti, S. M., Lombardi, L., Ricciardi, P., Morelli, M., Candelori, C., & Babore, A. (2021). COVID‐19 and home confinement: A study on fathers, father–child relationships and child adjustment. *Child: Care, Health and Development*. Advance online publication. 10.1111/cch.12912 10.1111/cch.12912 PMC8653319 34510515

[CR68] Van Ouytsel J, Punyanunt-Carter NM, Walrave M, Ponnet K (2020). Sexting within young adults’ dating and romantic relationships. Current Opinion in Psychology.

[CR69] Van Ouytsel J, Van Gool E, Walrave M, Ponnet K, Peeters E (2017). Sexting: Adolescents’ perceptions of the applications used for, motives for, and consequences of sexting. Journal of Youth Studies.

[CR70] Van Ouytsel J, Walrave M, Ponnet K (2019). Sexting within adolescents' romantic relationships: How is it related to perceptions of love and verbal conflict?. Computers in Human Behavior.

[CR71] Vergés Bosch, N., Freude, L., Camps Calvet, C., & Collado Sevilla, A. A. (2021). Aprendizaje Servicio, Género y TIC. De la desigualdad de género en las TIC a la generación de vocaciones tecnológicas en el ámbito educativo. *Foro de Educación, 19*(2), 221–243. 10.14516/fde.885

[CR72] Walker S, Sanci L, Temple-Smith M (2013). Sexting: Young women's and men's views on its nature and origins. Journal of Adolescent Health.

[CR73] Wekerle C, Wolfe DA (1999). Dating violence in mid-adolescence: Theory, significance, and emerging prevention initiatives. Clinical Psychology Review.

[CR74] Weden MM, Zabin LS (2005). Gender and ethnic differences in the co-occurrence of adolescent risk behaviors. Ethnicity and Health.

[CR75] Wignall L, Portch E, McCormack M, Owens R, Cascalheira CJ, Attard-Johnson J, Cole T (2021). Changes in sexual desire and behaviors among UK young adults during social lockdown due to COVID-19. The Journal of Sex Research.

[CR76] Wijayanti, Y. T. (2021). Long distance marriage couple communication pattern during the Covid-19 pandemic. *Journal ASPIKOM, 6*(1), 208–221. 10.24329/aspikom.v6i1.718

[CR77] Wolak J, Finkelhor D, Mitchell KJ (2012). How often are teens arrested for sexting? Data from a national sample of police cases. Pediatrics.

[CR78] Worthen MG, Lingiardi V, Caristo C (2017). The roles of politics, feminism, and religion in attitudes toward LGBT individuals: A cross-cultural study of college students in the USA, Italy, and Spain. Sexuality Research and Social Policy.

[CR79] Woznicki N, Arriaga AS, Caporale-Berkowitz NA, Parent MC (2020). Parasocial relationships and depression among LGBQ emerging adults living with their parents during COVID-19: The potential for online support. Psychology of Sexual Orientation and Gender Diversity.

